# Magnetic Resonance Elastography of Upper Trapezius Muscle

**DOI:** 10.1002/nbm.70007

**Published:** 2025-02-26

**Authors:** Emi Hojo, Wiraphong Sucharit, Saranya Jaruchainiwat, Punthip Thammaroj, Julaluck Promsorn, Prathana Chowchuen, Kevin J. Glaser, Uraiwan Chatchawan, Neil Roberts

**Affiliations:** ^1^ Centre for Reproductive Health (CRH), Institute for Regeneration and Repair (IRR), Edinburgh BioQuarter University of Edinburgh Edinburgh UK; ^2^ School of Physical Therapy, Faculty of Associated Medical Sciences (AMS) Khon Kaen University (KKU) Khon Kaen Thailand; ^3^ Research Centre in Back, Neck, Other Joint Pain and Human Performance (BNOJPH) Khon Kaen University Khon Kaen Thailand; ^4^ Department of Radiology, Faculty of Medicine Khon Kaen University Khon Kaen Thailand; ^5^ Department of Radiology, Mayo Clinic College of Medicine Mayo Clinic Rochester Minnesota USA

**Keywords:** actuator, caliper technique, magnetic resonance elastography (MRE), massage, stiffness, upper trapezius (UT) muscle, vibration frequency

## Abstract

The goal of the present study was to investigate the effect of positioning a soft flexible tube‐based actuator parallel or orthogonal to the principle muscle fibre direction, on measurements of the stiffness of upper trapezius (UT) muscle obtained using magnetic resonance elastography (MRE). The effects of using three different vibration frequencies (60 Hz, 80 Hz and 100 Hz) and studying left and right sides of the body were also investigated. The relevant MRE datasets were acquired on a 1.5 T MRI system using a 2D gradient‐echo (GRE) MRE sequence, and corresponding wave images produced using multimodel direct inversion (MMDI) were analysed by two observers using the manual caliper technique. Except for two of the 108 individual datasets, when the agreement was moderate, there was substantial to perfect agreement between wave quality scores obtained by the two observers, with an identical mean value. Similarly, and again with only two exceptions, there was good to excellent agreement between the measurements of UT stiffness obtained by the two observers. UT stiffness values obtained when the acoustic waves were propagating along the principle muscle fibre direction were significantly higher than when the waves were propagating orthogonal to the principle muscle fibre direction at all vibration frequencies (*p* < 0.005), and only for the former was a significant dispersion effect observed whereby stiffness increased as frequency increased (*p* < 0.05). No significant asymmetry was observed in measurements of UT stiffness obtained for the left and right sides of the body (*p* = 0.29). In conclusion, the new soft and flexible tube‐based actuator is comfortable and produced very good wave propagation in UT when positioned in either orientation. However, it is recommended for wave propagation to be induced in the principle fibre direction and there was found to be no advantage in using a vibration frequency above 60 Hz.

## Graphical abstract

A new MR elastography protocol was developed for measuring stiffness of upper trapezius (UT) and the effects of actuator placement (vertical and horizontal), vibration frequency (60, 80 and 100 Hz) and body side (left versus right) were investigated. The illustration shows muscle stiffness being obtained by measuring the wavelength of acoustic waves with 80‐Hz vibration frequency propagating from the actuator placed vertically and adjacent to the neck on the right shoulder.

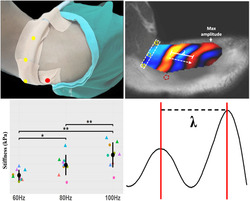

**Abbreviation**
UT: upper trapezius
OS: office syndrome
PT: physical therapy
MRE: magnetic resonance elastography
VAP: vertical actuator position
HAP: horizontal actuator position
SNR: signal‐to‐noise ratio
ROI: region of interest
REC: Research Ethics Committee
MT: middle trapezius
C7: seventh cervical vertebra
MEG: motion‐encoding gradient
F‐H: foot‐to‐head
MMDI: multimodel direct inversion
SD: standard deviation
ICC: intra‐class correlation coefficient
LoA: limits of agreement
UB: upper bound
LB: lower bound
CI: 95% confidence interval
CV: coefficient of variation
US‐SWE: ultrasound shear wave elastography
DTI: diffusion tensor imaging
TI‐NLI: transverse isotropic implementation of subzone‐based nonlinear inversion
2D GRE: two‐dimensional gradient‐echo
μ ROI: mean signal intensity in the region of UT muscle
σ bg: standard deviation of signal intensity in a background ROI

## 1 Introduction

Upper trapezius (UT) is the most frequently affected muscle in patients experiencing neck and shoulder pain, and previous studies using magnetic resonance elastography (MRE) [1], which allows non‐invasive measurement of the mechanical properties of tissues [2, 3, 4, 5], have shown the stiffness of UT to be significantly increased in patients [2, 3, 4]. A crucial component of muscle MRE studies is the human–machine interface, whereby acoustic‐frequency shear waves are induced in the muscle of interest, by means of vibrations produced using a purpose‐built actuator and imaged by using an appropriate pulse sequence. Clinical MRE protocols have been developed for studying the liver, with diagnosis of hepatic fibrosis based on analysis of elastograms (i.e. so‐called stiffness maps) that are computed automatically using the information in the wave images. However, the elastograms must be analyzed with caution in the case of skeletal muscle which is inherently anisotropic. Debernard et al. [6] have demonstrated that in the case of muscle the propagation of the shear waves typically occurs along the direction of the muscle fibres and that muscle stiffness can be optimally obtained by measuring wavelength along the principle fibre direction using an electronic caliper [5, 7, 8, 9].

UT is the most superior region of the sheet‐like kite‐shaped trapezius muscle that is oriented laterally from the base of the skull across the shoulders to the acromion and ends inferiorly at the middle of the thoracic vertebrae. In the MRE studies of UT performed by Chen and co‐workers [2, 3, 4], subjects lay prone and the muscle was vibrated using a pneumatically driven actuator positioned adjacent to the neck on one side of the body and which generated waves that propagated medially to laterally in UT. Ito et al. [5] used an alternative approach in which subjects lay supine and on top of an appropriately padded 5 cm × 5 cm pneumatic actuator, made of hard plastic, attached to the skin above the posterior‐distal part of the shoulder and which allowed simultaneous study of trapezius and supraspinatus muscles.

Patients who are experiencing severe neck and shoulder pain may find it uncomfortable to lie prone in the MRI system for an investigation that may take 30 min. Therefore, in the present study, the subjects lay supine in the MRI system and two different positions of a soft and flexible tube‐based pneumatic actuator were investigated. Firstly, similar to the previous MRE studies by Chen and co‐workers [2, 3, 4, 5], but with the subject now lying supine, by placing the actuator adjacent to the neck, shear waves were induced in UT that propagated parallel to the principal muscle fibre direction [10]. Secondly, the tube‐based actuator was rotated by 90° and placed along the anterior margin of the clavicle, so that the propagating waves ran from the actuator down the back in an oblique anterior to posterior direction, orthogonal to the principal fibre direction. The well‐fitting position of the actuator in a natural recess of the body adjacent to the clavicle provided increased comfort, however, it is unusual for muscle MRE studies to be performed in which waves are induced in a direction other than along the principal fibre orientation. Stiffness values are known to be dependent on the angle between the wave propagation direction and the principal muscle fibre direction and are reported to be highest when waves propagate along the muscle fibre direction [11, 12], and these effects needed to be investigated before the actuator could be used in clinical research to study patients with neck and shoulder pain.

Another important consideration in any MRE study is vibration frequency. Low‐frequency waves will penetrate deeper in the body than high‐frequency waves and typically an MRE frequency of 60 Hz is used to measure liver stiffness in clinical practice [13]. However, high‐frequency waves can be readily induced in the relatively superficial UT muscle and a vibration frequency of 75 Hz was used by Ito et al. [5], and a frequency of 150 Hz by Chen et al. [3, 4]. In the present study, with the aim of developing a standardised muscle MRE protocol that could be used in clinical research, the effect of using vibration frequencies of 60 Hz, 80 Hz and 100 Hz was investigated.

There are three potential factors relating to dimensionality to be considered in describing the setup of an MRE experiment, namely (i) the orientation and number of directions of the magnetisation encoding gradient (MEG) used to measure the tissue displacements produced by the propagating waves, (ii) whether the data refer to a profile, a slice or a volume and (iii) whether the inversion algorithm is applied separately to consecutive image sections or to several sections at once (i.e. processing for a section includes information from the sections above and below). However, the use of the expressions 2D MRE and 3D MRE which are in common use have a more nuanced meaning [14]. In particular, in using the description 2D MRE, there is an implicit assumption that the direction of the propagation of the shear waves produced by the actuator is contained within the tissue section that is being imaged, and the MEG is applied perpendicular to the section, whereas in the case of 3D MRE the algorithms that are used to process the data solve the wave propagation in all three directions. On the other hand, 1D MRE refers to the situation when the direction of wave propagation is identified on images that have typically been obtained using 2D MRE and an analysis is performed for a selected profile chosen along this direction using an electronic caliper, as in the present study, or perhaps using phase gradient analysis [15] as will be referred to later.

In the present study, 2D GRE MRE data were acquired for UT muscle in nine healthy young subjects using a soft and flexible tube‐based actuator placed in two different positions so as to produce shear waves that ran parallel and orthogonal to the principle muscle fibre direction, respectively, at three different vibration frequencies (60 Hz, 80 Hz and 100 Hz), and on both sides of the body. Data were analysed using the 1D manual caliper technique in three stages with the following objectives. Firstly, the signal‐to‐noise ratio (SNR) of the images was measured. Secondly, by obtaining repeat measurements by two independent observers the reproducibility of the subjective assessment of wave quality score and measurement of UT stiffness was investigated. Thirdly, whether there were statistically significant effects of actuator position relative to muscle fibre direction, vibration frequency and side of the body were investigated.

## 2 Materials and Methods

### 2.1 Participants

The study was approved by the Research Ethics Committee (REC) of Khon Kaen University (Reference: HE631017, Thai Clinical Trials Registry: TCTR20200805001). Nine healthy subjects (four females and five males, mean age 28.7 ± 6.8 years) were recruited and provided fully informed written consent of their willingness to participate. All participants satisfied the study criteria including BMI ≥ 30 kg/m^2^, severity of neck or shoulder pain in UT muscle over the past 12 weeks that is at a level equal to or greater than 3 cm as measured using a subjective visual analogue scale with range 0 to 10 cm, no diagnosis of myofascial trigger points, no restriction in cervical range of motion, and no other concurrent relevant medical diagnosis.

### 2.2 Experiment

#### 2.2.1 Actuator Setup

**FIGURE 1:**
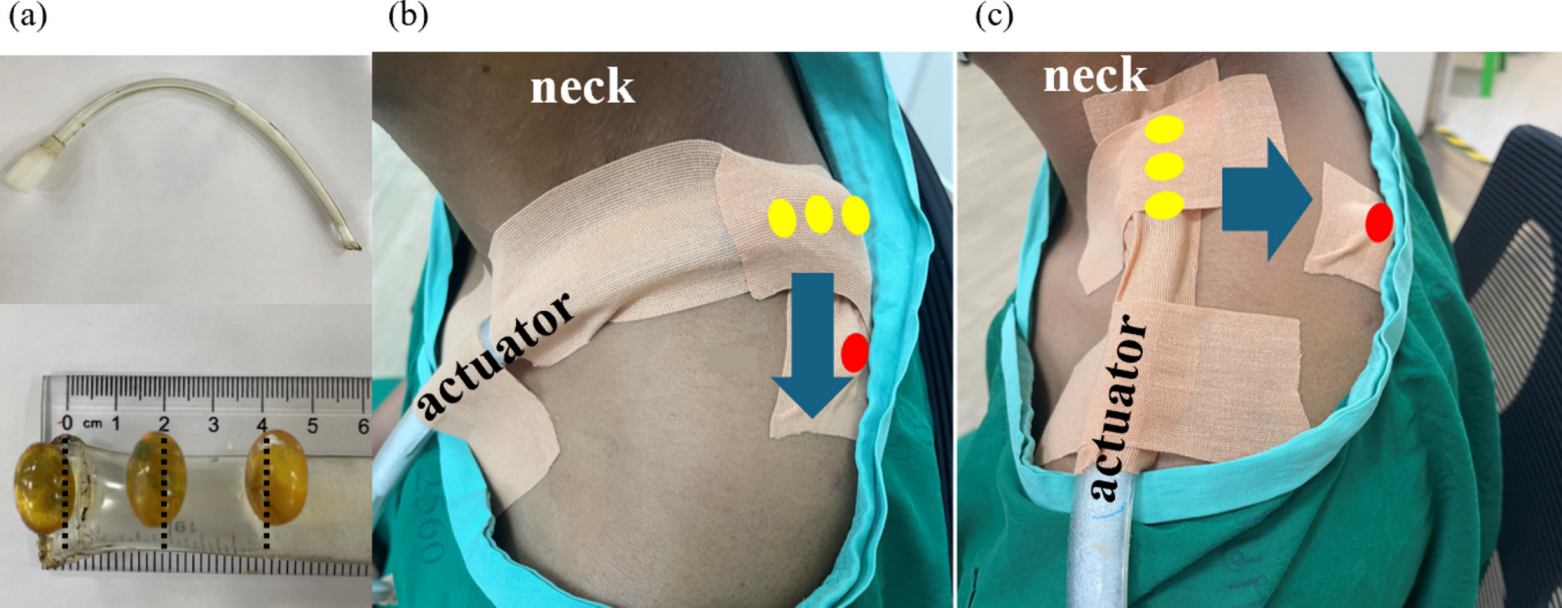
Illustration of the position of the (a) soft and flexible tube actuator to which are attached three fish oil capsules, with a 2 cm space between each capsule, in (b) vertical (VAP) and (c) horizontal (HAP) positions. In (b) and (c), the fish oil capsules attached to the actuator are denoted by yellow symbols, the point on the border of upper trapezius (UT) and the middle trapezius (MT) halfway between C7 and the acromion process is marked by a fish oil capsule denoted by a red symbol and blue arrows indicate the main direction of MRE wave propagation.

The actuator consists of a plastic tube that is sealed and flattened into a rectangular profile and slightly padded with mesh material at the end which is in contact with the body (1.6 cm outer diameter, 0.16 cm wall thickness, 91.5 cm length, Mayo Clinic, Rochester, Minnesota, USA) (Figure 1a). Three fish oil capsules were attached with stretchable tape consecutively on top of the actuator at approximately 2 cm intervals, beginning 2  cm from the tip of the actuator, so that the portion of the actuator that was in contact with the skin could be seen on the MR images (Figure 1b,c). In addition, to facilitate identification on the MR images, a fish oil capsule was placed with medical‐grade, double‐sided, adhesive tape on the border between UT and middle trapezius (MT) muscle halfway between the seventh cervical vertebra (C7) and the acromion process, and stretchable tape was applied to fix the capsule in place.

The actuator with fish oil capsules attached was taped to the participant and in conformity with the shape of their shoulders in the two different orientations in turn: i.e. either in a vertical actuator position [VAP] in which case waves will be generated that propagate in a direction parallel to the principle direction of the muscle fibres in UT, or in a horizontal actuator position [HAP] in which case waves will be generated that propagate in a direction orthogonal to the principle direction of the muscle fibres in UT (see Figure 1). In order to make a good contact between the actuator and the surface of the skin, and to target the vibration in UT, a 15 cm length of moderately stretched Kinesio tape was applied along the actuator and additionally two half lengths (7.5 cm) of the Kinesio tape were applied in an orthogonal direction to hold the actuator in place. Finally, an elastic bandage (10 cm × 4.5 m) was placed over the actuator and wrapped around the shoulder in a manner that was sufficiently loose so as not to compress the soft tissue underneath. This procedure was performed by the same experienced physical therapist (WS) for all participants prior to MRI scanning. Left and right UT were studied in random order.

#### 2.2.2 MRI and MRE Image Acquisition

**FIGURE 2:**
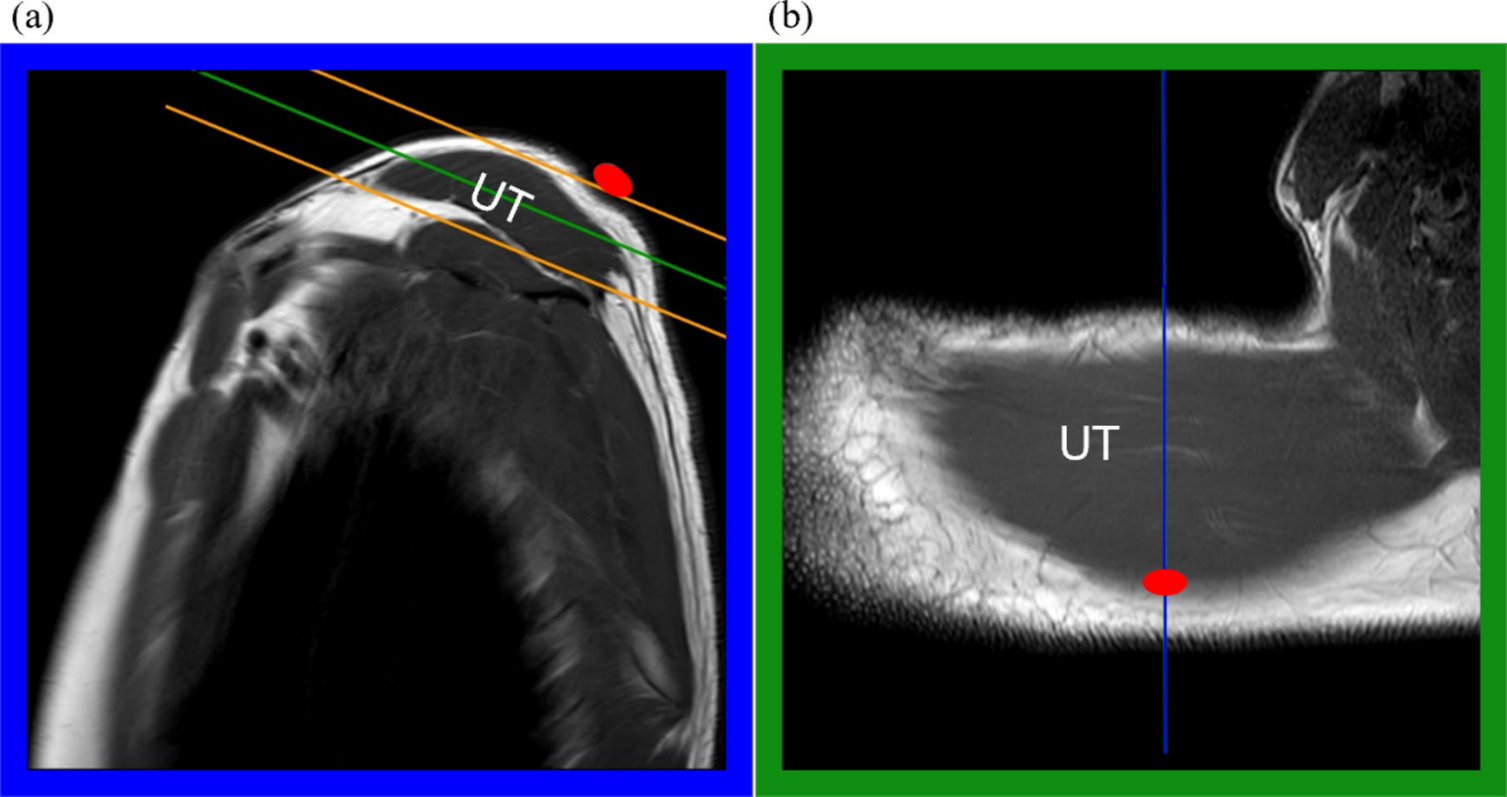
Localisation of UT in the MR images. The blue line in (b) corresponds to the position of the sagittal section in (a). A series of oblique axial images were prescribed to cover the entire UT muscle between the orange lines in (a). The green line in (a) corresponds to the position of the oblique axial section of UT in (b) which is obtained at a depth of approximately 1 cm beneath the subcutaneous tissue. The position of the border between UT and MT is marked by the fish oil capsule denoted by a red symbol.

All MRI and MRE datasets were acquired using a 1.5 T MRI system (Ingenia Ambition X, Philips Healthcare, Best, The Netherlands) equipped with a 16 channel extremity MRI shoulder coil (Shoulder Coil 16, Philips Healthcare, Best, The Netherlands). First, a series of three orthogonal localiser MR images of UT were acquired with a 2D turbo‐spin‐echo MRI sequence from which the MRE images were prescribed. In particular, the anatomical reference point on the border between UT and MT was identified on the sagittal localiser images by means of the fish oil capsule (Figure 2a) and used to guide the prescription of a series of axial oblique MRE images through UT muscle (Figure 2b).

With the participant lying supine in the MRI system, the actuator was connected via a long hose to an active driver (Resoundant Inc., Rochester, Minnesota, USA) which is the source of the acoustic waves and synchronised with the MRI system computer, and separate MRE datasets were acquired for VAP and HAP actuator positions with vibration frequencies of 60 Hz, 80 Hz and 100 Hz for both sides of the body using a 2D gradient‐echo (GRE) MRE sequence. The motion‐encoding gradient (MEG) was applied in the foot‐to‐head (F‐H) direction orthogonal to the section. Pairs of magnitude and phase images were acquired for each of four phase offsets with TR 50 ms, TE 20.54 ms, flip angle 20°, FOV 18 cm × 18 cm, pixel matrix 128 × 128 and slice thickness 5 mm in an acquisition time of 1.15 min. The four phase images were processed using the multimodel direct inversion (MMDI) post‐processing algorithm to produce elastograms [16]. Several types of inversion algorithm have been reported in the literature, including local frequency estimation [17, 18, 19], multiscale direct inversion (MSDI) [20, 21, 22, 23] and multimodel direct inversion (MMDI) [16, 24]. MMDI has been reported to provide several improvements over MSDI, including superior resolution, less noise and shorter processing time [25]. Although stiffness maps produced by the MMDI algorithm appeared on the MRI system console, they were not analysed in the present study. Instead, the analysis was focussed on a series of eight images that were also produced in the process of applying the algorithm and which have an enhanced appearance compared to the original phase images. The latter are referred to as wave images and can be animated to allow convenient dynamic visualization of the wave propagation in the tissue of interest. It is these waves images that were analysed by using the caliper technique as will be described below.

### 2.3 Image Analysis

#### 2.3.1 Measurement of SNR for UT Magnitude Images

**FIGURE 3:**
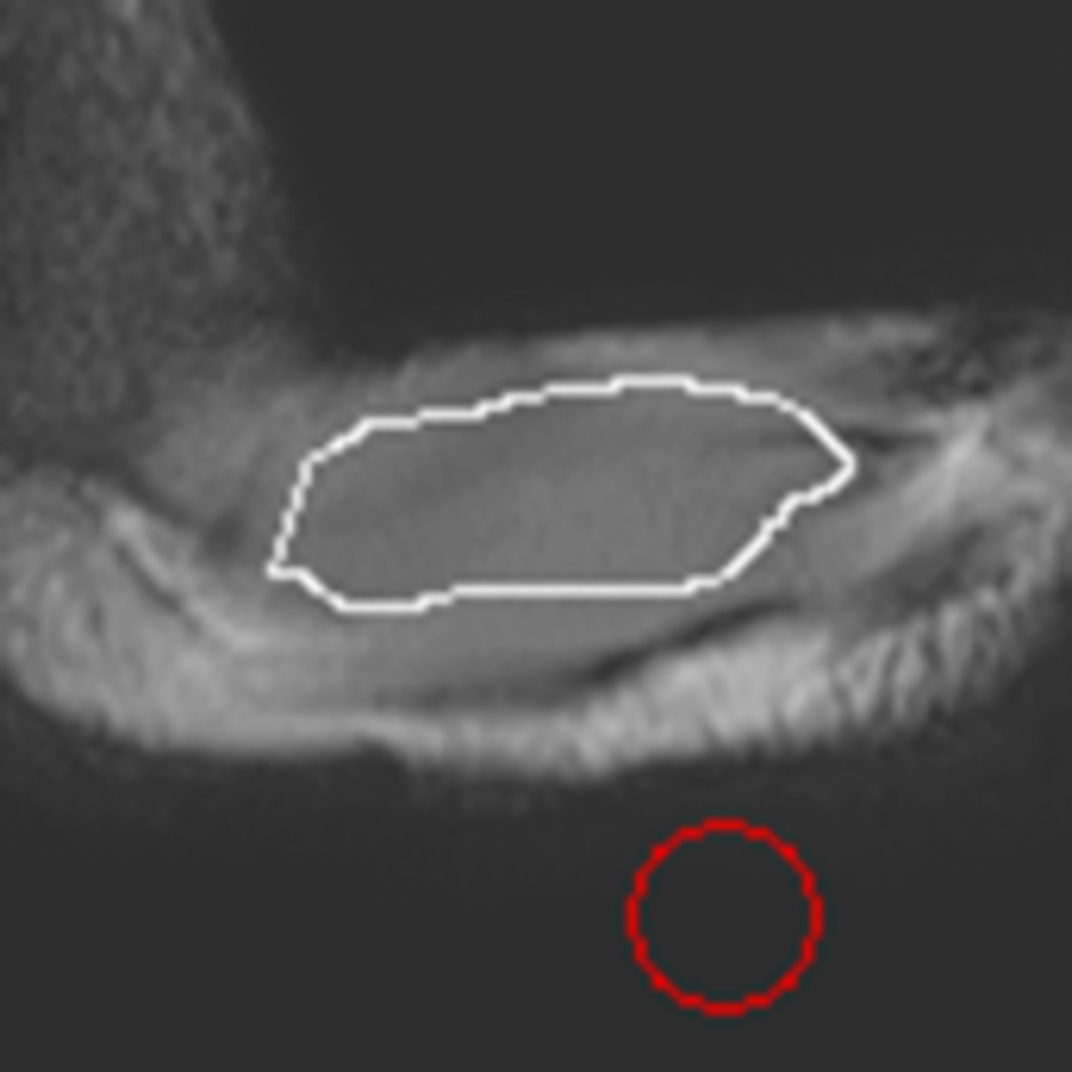
UT (white outline) and background (red circle) were delineated on the MRE magnitude image to measure signal and noise, respectively.

Image quality was assessed by computing SNR [15] on the MRE magnitude images using MRE‐Lab software (Mayo Clinic, Rochester, Minnesota, United States). In particular, mean signal intensity (S) in the region of UT muscle ROI_m_ (see white outline in Figure 3), and noise corresponding to the standard deviation (SD) of the signal intensity (σ) in a background ROI_bg_ (see red circle in Figure 3) were measured and entered in the following Equation [1], where the factor 0.655 is a correction to the standard equation to take account of the signal in the background having a Rician rather than Gaussian distribution:

(1) $$\mathrm{SNR}=0.655*\frac{S\;{\mathrm{ROI}}_{m}}{\sigma \;{\mathrm{ROI}}_{\mathrm{bg}}}$$

#### 2.3.2 Wave Quality Score Measurement

**FIGURE 4:**
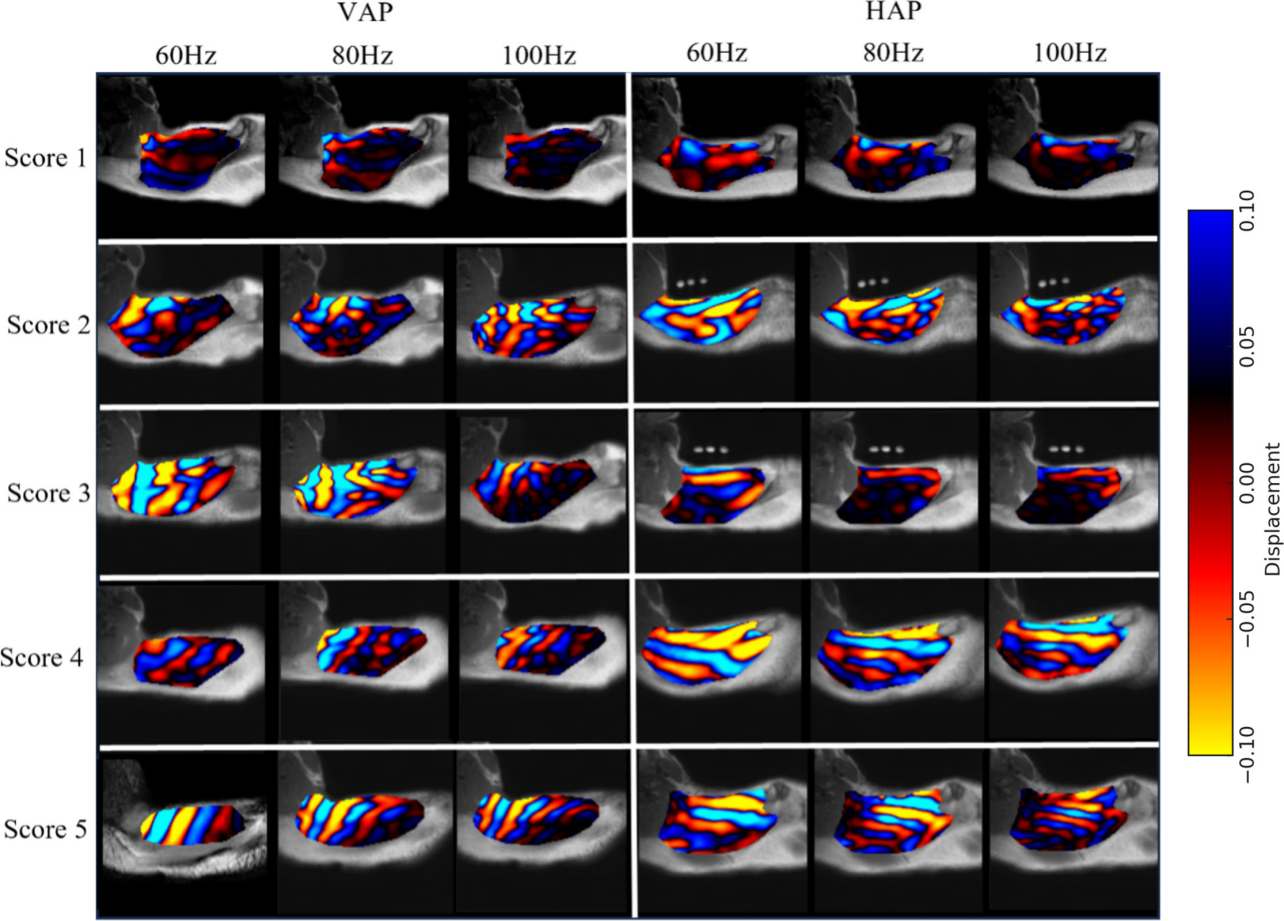
Example wave images overlain on corresponding MRE magnitude images of UT for VAP, in the three left‐hand columns, and for HAP, in the three right‐hand columns, obtained at vibration frequencies of 60 Hz, 80 Hz and 100 Hz. Examples of wave quality scores of 1 to 5 (see text) are illustrated from top to bottom. Wave propagation is from left to right for VAP and top to bottom for HAP. The change from blue to red colour in the wave images corresponds to half a wavelength. The scale on the colour bar corresponds to wave amplitude in arbitrary units.

Assessment of wave quality score was performed using MRE‐Quant software (Mayo Clinic, Rochester, Minnesota, USA) according to a five‐point scale proposed by Ito et al. [5] (Figure 4).

A score of 1 corresponds to when no visibly coherent waves, and a score of 2 when no planner waves can be seen within UT. A score of 3 corresponds to when wave propagation was clearly visible with less than one wavelength within UT. A score of 4 corresponds to when wave propagation was clearly visible with more than one and less than two wavelengths within UT, and a score of 5 corresponds to when wave propagation was clearly visible with two or more wavelengths within UT. In the present study, wave quality score was recorded for the wave image that showed the clearest and most extensive wave propagation among the series of eight wave images.

#### 2.3.3 UT Stiffness Measurement

**FIGURE 5:**
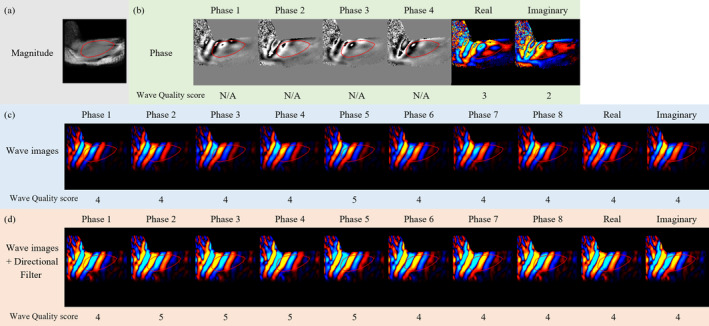
Example of MRE magnitude image (a) for UT together with corresponding phase images (b), wave images (c) and wave images after processing with a directional filter (DF) in the direction of wave propagation (d) at 60 Hz. Phases 1 to 8 correspond to the phase offset image number. Real and Imaginary refer to the real and imaginary parts of the first harmonic of the FT of the respective phase data (b), wave data (c) and DF wave data (d). The red outline refers to the UT region of interest (ROI). Values of wave quality score are noted for each phase of the wave images beneath the corresponding image.

The caliper technique has been applied using slightly different approaches in several previous publications. Firstly, Bensamoun et al. [7] obtained measures of wavelength for each of the phase images that had been acquired (see panels labelled phase 1 to 4 in Figure 5b). Secondly, Chakouch et al. [26] applied a directional filter (DF) [27] in the direction of the wave propagation and obtained measures of wavelength for the filtered wave images (see panels labelled phase 1 to 8 in Figure 5d). Thirdly, Bensamoun et al. [28] applied a temporal Fourier transform (FT) and obtained measures of wavelength from the real and imaginary components of the FT (see Figure 5b).

**FIGURE 6:**
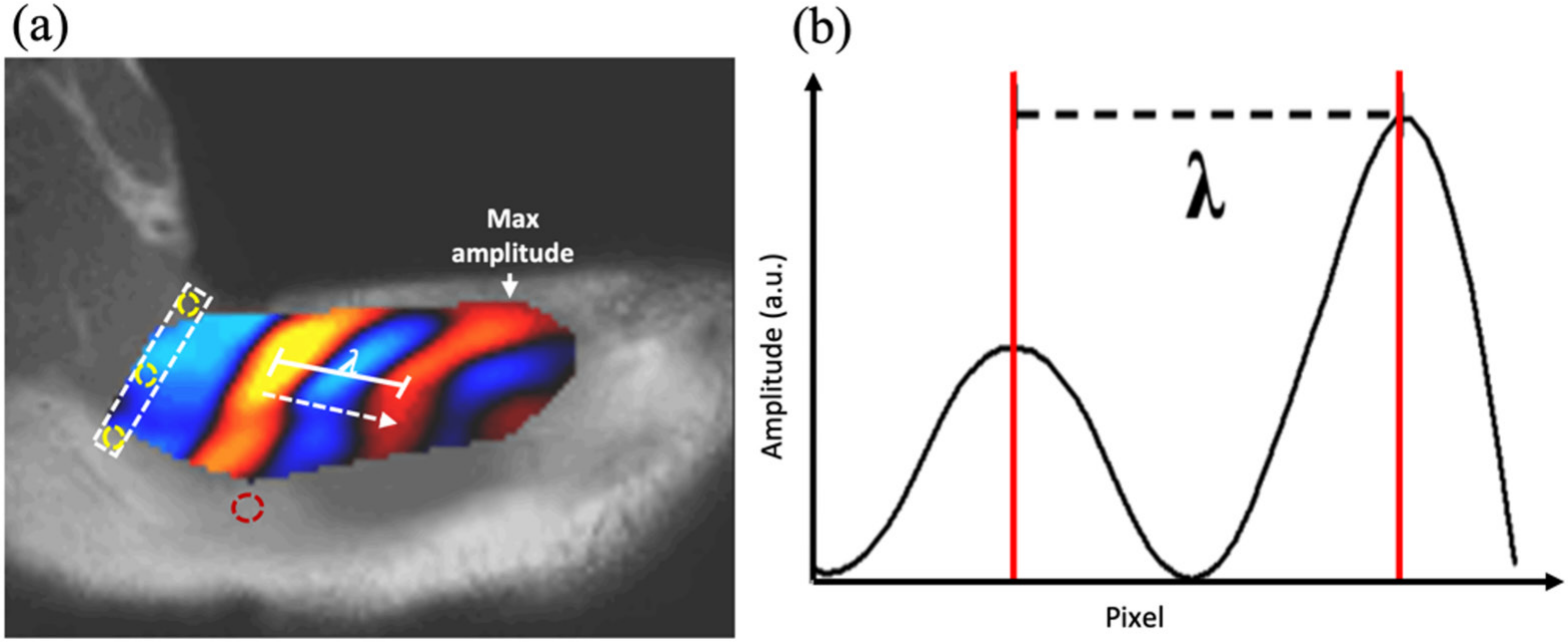
(a) Image of a propagating wave in UT obtained at a vibration frequency of 80 Hz overlaid on the corresponding magnitude image. The white rectangle represents the position of the VAP actuator with fish oil capsule markers represented by yellow circles. The red circle denotes a fish oil capsule which marks the border between upper and middle trapezius muscle, and the dashed arrow denotes the direction of wave propagation. The red vertical lines that intersect the profile of the wave amplitude trace shown in (b) are drawn at the positions of the end points of the white line segment shown in (a) and correspond to one full wavelength λ.

In the present study, the caliper technique was applied after the wave images had been processed to reduce noise, and after a bandpass filter and interpolation had been applied, but without a DF being applied. This is similar to the first of the approaches referred to in the paragraph above (i.e. Bensamoun et al. [27]) and was the approach also used by Numano et al. [9] and Ito et al. [5, 29] The process was performed using MRE‐Lab software (Mayo Clinic, Rochester, Minnesota, United States), and measurements of wavelength were obtained by applying the caliper technique to a 1D profile drawn along the most obvious direction of wave propagation in the muscle. In particular, whilst viewing an animation of the propagating waves within the UT, the observer drew a 1D profile along the direction in which the waves were propagating. The animation was then stopped, and an electronic caliper was used to record the distance between the relevant peaks or troughs for the eight wave images (Figure 6).

The wavelengths measured by the caliper technique for each wave image with wave quality score of 3 or above were averaged, and UT stiffness was obtained by substituting the value in Equation (2).

(2) $$\mu =\rho {\left(\mathrm{\lambda f}\right)}^{2}$$

where ρ is the tissue density (assumed to be 1000 kg/m^3^), λ is the wavelength (in metres), and f is the vibration frequency (Hz).

#### 2.3.4 Data Analysis

##### 2.3.4.1 SNR of UT Magnitude Images

For the relatively small region of UT (i.e. 2 cm by 2 cm) close to the actuator where both VAP and HAP produced good wave propagation, one of the observers (EH) drew an ROI on the MRE magnitude images obtained for each actuator position, vibration frequency and side of the body, and SNR was calculated according to the procedure described in Section 3.1 above.

##### 2.3.4.2 Inter‐Observer Reproducibility Study of Wave Quality Score and UT Stiffness

To measure inter‐observer reproducibility, two observers (EH and WS), who both have 2 years of experience in muscle MRE data analysis, made a single assessment of wave quality score and applied the caliper technique to measure UT stiffness once for the total of 108 MRE datasets which refer to two actuator positions (VAP, HAP), three vibration frequencies (60 Hz, 80 Hz and 100 Hz), and two sides of the body (left and right) for the nine healthy participants.

### 2.4 Statistical Analysis

Statistical analyses were performed using R software (Version 1.3.1093 © 2009–2020 Rstudio, PBC) with significance level set to *p* < 0.05.

#### 2.4.1 SNR Measurements for UT on the MRE Magnitude Images

The mean and SD of the SNR measurements obtained for UT in the magnitude images corresponding to the 108 datasets obtained for the nine participants were analysed using a three‐way repeated measures ANOVA to assess whether there were any significant effects due to actuator position, vibration frequency or side of the body.

#### 2.4.2 Inter‐Observer Reproducibility of Wave Quality Score

Inter‐observer reproducibility of the wave quality scores obtained by the two observers for the 108 datasets, representing two different actuator positions, three different vibration frequencies and both sides of the body, was assessed by using Cohen's kappa coefficient. When the value of Cohen's kappa coefficient is 0 agreement is rated as poor, for values 0 to 0.20 as slight, 0.21 to 0.40 as fair, 0.41 to 0.60 as moderate, 0.61 to 0.80 as substantial, and 0.81 to 1 as almost perfect (see Ito et al. [5]).

#### 2.4.3 Inter‐Observer Reproducibility of UT Stiffness

Inter‐observer reproducibility of the 108 UT stiffness measurements obtained by the two observers was assessed by using the intraclass correlation coefficient (ICC) [30] with a two‐way mixed model. In addition, by using the approach of Bland and Altman [31], the mean and difference of the UT stiffness measurements obtained by the two observers were calculated for the two different actuator positions, three different vibration frequencies and both sides of the body. The bias, known as systematic error, and the SD of the bias were computed. The limits of agreement (LoA) was then computed as 1.96 times the SD, and the upper bound (UB) and lower bound (LB) of the 95% confidence interval (CI) were also computed [31]. In addition, the coefficient of variation (CV), defined as the ratio of the SD to the overall mean of the two UT stiffness measurements, was calculated and converted to a percentage to assess variability due to actuator position, vibration frequency and side of the body.

#### 2.4.4 Effect of Actuator Position, Vibration Frequency and Side of the Body on Wave Quality Score

Friedman's test was used to assess whether the mean, maximum and minimum values of the wave quality score obtained by the two observers for the 108 datasets showed significant effects with respect to actuator position, vibration frequency or side of the body.

#### 2.4.5 Effect of Actuator Position, Vibration Frequency and Side of the Body on UT Stiffness

Friedman's test, and subsequent post hoc analysis using a pairwise Wilcoxon test with Bonferroni correction, was used to assess whether the measurements of UT stiffness obtained by the two observers for the 108 datasets showed significant effects with respect to actuator position, vibration frequency or side of the body.

## 3 Results

### 3.1 SNR for UT on MRE Magnitude Image

**FIGURE 7:**
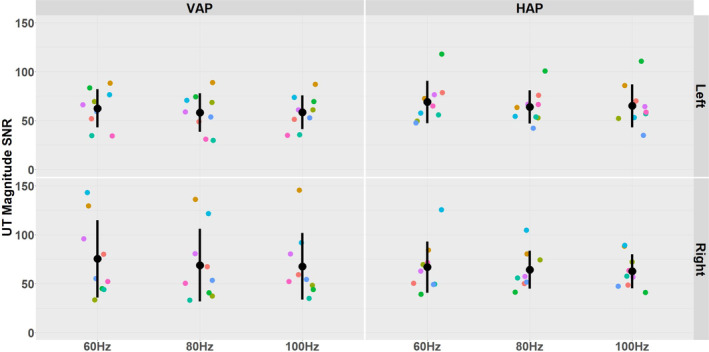
Distribution of SNR measurements of UT are shown for the nine subjects for VAP (left panels) and HAP (right panels) actuator position, three different vibration frequencies (60 Hz, 80 Hz and 100 Hz) on the left (top row) and right (bottom row) sides of the body. The error bars show the mean and SD. The unique colours of the data points refer to individual participants.

The mean and SD of the SNR measurements obtained for UT in the MRE magnitude images acquired for different actuator position, vibration frequency and side of the body are presented for the 108 datasets in Figure 7.

Results of a three‐way repeated measures ANOVA test showed no significant difference in the measurement of SNR with respect to actuator position, vibration frequency or side of the body (*p* > 0.05).

### 3.2 Inter‐Observer Reproducibility of Wave Quality Score

Values of Cohen's kappa coefficient for the agreement between the wave quality scores obtained by the two observers for the 108 datasets representing two actuator positions, three vibration frequencies and two side of the body are presented in Table 1.

**TABLE 1:** A total of 108 mean wave quality scores and 108 UT stiffness measurements acquired for two actuator positions (VAP, HAP), three vibration frequencies (60 Hz, 80 Hz and 100 Hz) and both sides of the body (left and right) for the nine healthy participants. Cohen's kappa (kappa) for inter‐observer reproducibility of UT wave quality score and ICC, bias, and the upper (UB) and lower bound (LB) of the limits of agreement (LoA) for inter‐observer reproducibility of UT stiffness measurements are presented. Coefficient of variation (CV) refers to the ratio of SD and the mean value of the two UT stiffness measurements obtained by the two observers expressed as a percentage. Stiffness, bias and LoA values are presented as mean ± SD in kilopascals (kPa).

	Left							Right						
	Mean wave quality score	Kappa	Stiffness (kPa)	ICC	LoA (UB, LB) (kPa)	Bias (kPa)	CV (%)	Mean wave quality score	Kappa	Stiffness (kPa)	ICC	LoA (UB, LB) (kPa)	Bias (kPa)	CV (%)
	**VAP**													
60 Hz	4.11	0.83	4.25 ± 0.64	0.81	1.33 (1.92, −0.75)	0.59	22.5	4	0.80	4.22 ± 1.56	0.95	1.38 (1.33, −1.43)	−0.05	33.1
80 Hz	4.44	0.81	6.38 ± 1.92	0.94	1.92 (2.16, −1.68)	0.24	33.0	4.44	0.81	7.19 ± 2.97	0.94	2.68 (2.67, −2.69)	−0.01	40.0
100 Hz	4.44	0.78	8.35 ± 3.04	0.92	3.77 (4.13, −3.42)	0.36	41.7	4.56	0.58	10.1 ± 3.64	0.91	2.33 (4.15, −0.51)	1.82	36.3
	**HAP**													
60 Hz	4.11	0.57	1.92 ± 0.96	0.86	1.05 (1.33, −0.78)	0.27	43.7	4.33	0.65	1.68 ± 0.76	0.91	0.87 (0.80, −0.94)	−0.07	45.5
80 Hz	3.89	0.77	1.85 ± 0.86	0.07	2.47 (2.43, −2.50)	−0.04	31.7	4.44	0.80	1.81 ± 0.80	0.81	1.35 (1.24, −1.46)	−0.11	41.9
100 Hz	4.44	0.81	1.95 ± 0.62	0.59	1.34 (1.37, −1.31)	0.03	25.1	4.22	0.74	2.28 ± 1.09	0.87	1.33 (1.22, −1.44)	−0.11	41.4

For VAP, the values of Cohen's kappa coefficient showed substantial to almost perfect agreement between the wave quality scores recorded by the two observers, with the exception of right UT at 100 Hz when the agreement was moderate. For HAP, there was again substantial to almost perfect agreement between the wave scores recorded by the two observers, with the exception of left UT at 60 Hz, when the agreement was moderate.

### 3.3 Inter‐Observer Reproducibility of UT Stiffness

Regarding the agreement between UT stiffness measurements obtained by the two observers, for VAP there was good to excellent agreement for all vibration frequencies and on both sides of the body. For HAP, there was also good to excellent agreement between the stiffness measurements obtained by the two observers, with the exception of left UT at 80 Hz when the agreement was poor (ICC = 0.07).

**FIGURE 8:**
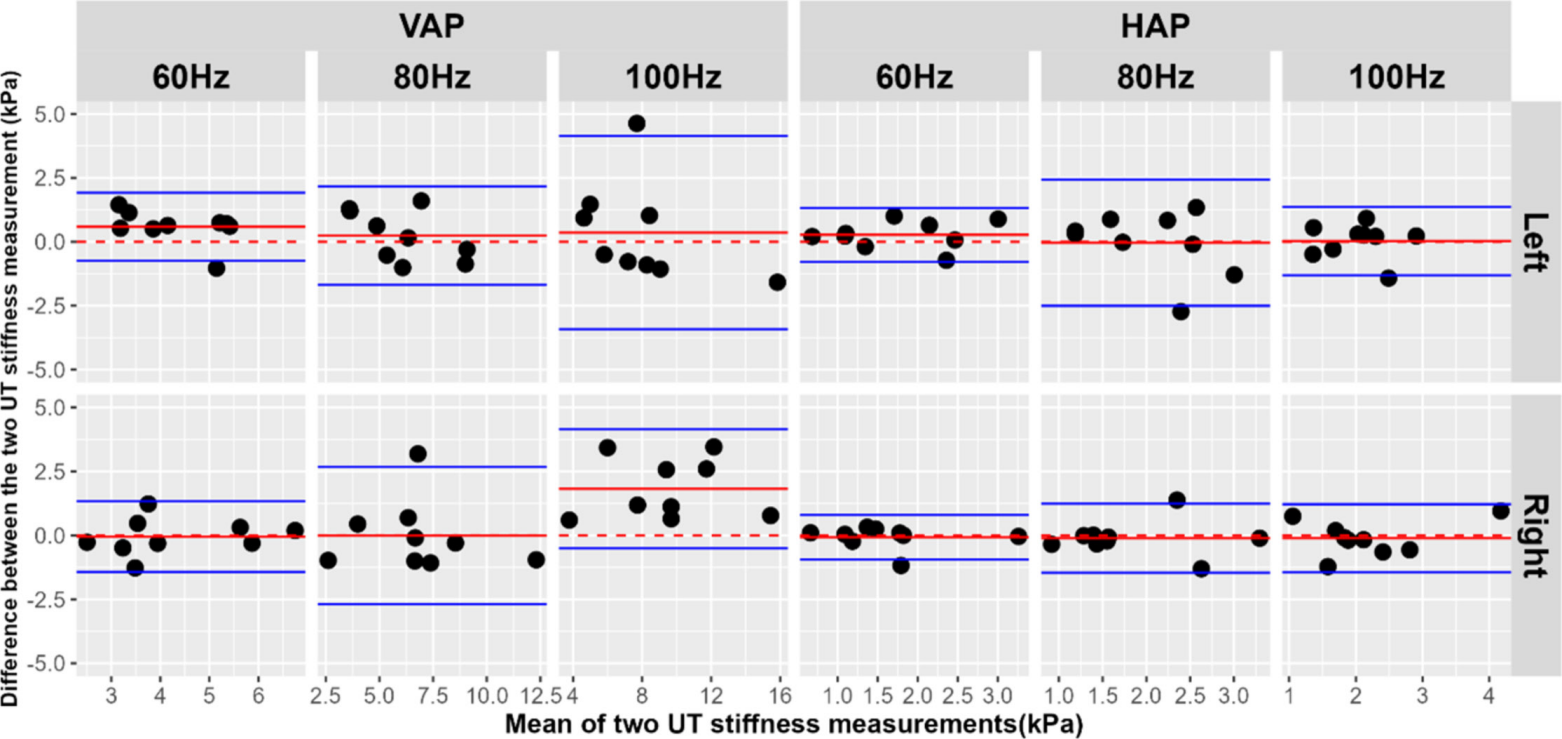
Bland–Altman plots of inter‐observer reproducibility of UT stiffness measurements with respect to actuator position (VAP in left column and HAP in right column), vibration frequency (60 Hz, 80 Hz and 100 Hz), and side of the body (left in top row and right in bottom row). The black circles represent the mean (horizontal axis) and difference (vertical axis) of the UT stiffness measurement obtained by the two observers. In each panel, the red lines correspond to the mean difference of UT stiffness values obtained between the two observers and the blue lines indicate the upper and lower values of the LoA. The dotted red line corresponds to zero and thus to no difference between the two measurements.

The results of the inter‐observer reproducibility studies performed for the 108 datasets analysed by both observers are presented as Bland–Altman plots with respect to actuator position, vibration frequency and side of the body in Figure 8. The LoA, UB, LB, bias (i.e. mean difference) as well as the CV of the two UT stiffness measurements obtained by the two observers for the different actuator positions, vibration frequencies and left and right side of the body are presented in Table 1.

The LoA accounts for both random and systematic errors between the two UT stiffness measurements [32], and for data points which plot outside the LoA, there is a 95% probability of there being a significant difference in the stiffness values measured by the two observers. In the case of VAP, LoA increased as vibration frequency increased, however, for HAP there was little variability in the values obtained across different frequencies. The bias (i.e. systematic error) can be considered negligible, except for the stiffness measurement obtained with VAP at 100 Hz for right UT with bias value of 1.82 kPa. CV values for the UT stiffness measurements obtained by the two observers ranged between 22.5% and 45.5% (see Table 1). The lowest variability was for VAP at 60 Hz and left UT when the CV was 22.5%, and the highest variability was for HAP at 60 Hz and right UT when the CV was 45.5%.

### 3.4 Effect of Actuator Position, Vibration Frequency and Side of the Body on Wave Quality Score

**FIGURE 9:**
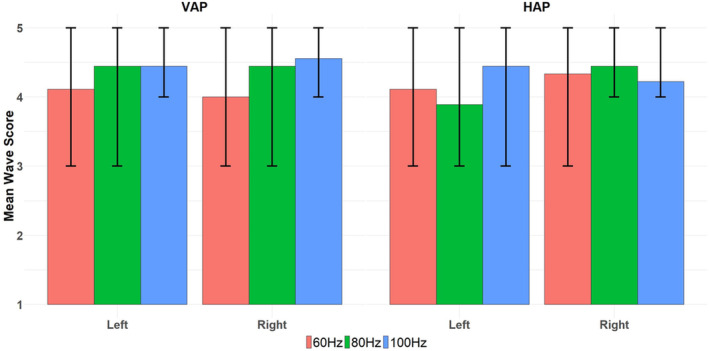
Mean wave quality scores for the 108 datasets acquired with different positions of the actuator (VAP in left column and HAP in right column) at three different vibration frequencies (60 Hz in orange, 80 Hz in green and 100 Hz in blue), and left and right sides of the body for the 9 participants. Values of the maximum and minimum score can be read from the black vertical lines in the centre of each column.

Mean, maximum and minimum values of wave quality score for the 108 datasets subdivided according to VAP and HAP, 60 Hz, 80 Hz and 100 Hz, and left and right UT side of the body are presented in Table 1 and plotted in Figure 9. Application of the Friedman test showed no significant difference in wave quality score with respect to actuator position, vibration frequency or side of the body (Friedman chi‐squared = 11.21, df = 11, *p* value = 0.43).

### 3.5 Effect of Actuator Position, Vibration Frequency and Side of the Body on UT Stiffness

**FIGURE 10:**
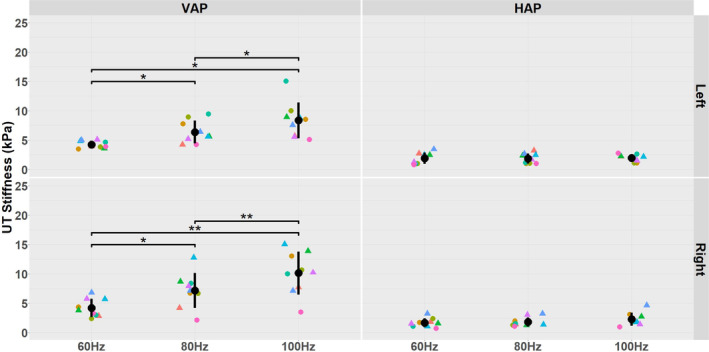
Distribution of UT stiffness measurements for the nine subjects is shown for the two different actuator positions, VAP (left column) and HAP (right column), three different vibration frequencies (60 Hz, 80 Hz and 100 Hz) and left and right sides of the body. The error bars correspond to the mean and SD of UT stiffness values. The unique colours of the data points refer to individual participants, and circle and triangle shapes refer to males and females, respectively. (*), and (**), indicate significance of *p* < 0.05 and 0.01, respectively.

UT stiffness measurements obtained using the caliper technique for the nine participants with respect to actuator orientation, vibration frequency and side of the body are presented in Table 1 and plotted in Figure 10.

Application of the Friedman test revealed there to be a significant interaction between UT stiffness and actuator position and vibration frequency (Friedman chi‐squared = 81.02, df = 11, *p* value = 9.373e−13), with no significant asymmetries (*p* > 0.05). Subsequent application of a post hoc pairwise Wilcoxon test with Bonferroni correction revealed that UT stiffness measured with VAP was significantly higher than with HAP at all vibration frequencies and for both left and right side of the body (*p* < 0.001). With regard to vibration frequency, in the case of VAP and for both left and right side of the body, the post hoc statistical pairwise Wilcoxon test with Bonferroni correction showed that UT stiffness was significantly higher at 100 Hz than at 80 Hz (*p* = 0.012 for left, *p* = 0.023 for right) and also at 100 Hz than at 60 Hz (*p* = 0.012 for left and *p* = 0.012 for right). Furthermore, for VAP and for both sides of the body, UT stiffness was significantly higher at 80 Hz than at 60 Hz (*p* = 0.012 for left, *p* = 0.035 for right). However, in the case of HAP and for both sides of the body, there was no significant effect of vibration frequency on UT stiffness (*p* > 0.05).

## 4 Discussion

The stiffness of UT muscle was measured in nine healthy participants using 2D GRE MRE for two different positions of a soft and flexible tube‐based actuator (VAP and HAP), three different vibration frequencies (60 Hz, 80 Hz and 100 Hz), and left and right sides of the body. With regard to the first objective of the study, measured SNR values were at least 20 for all participants which is consistent with a previous MRE study of the liver using the same 2D GRE MRE sequence [33]. Secondly, with only two exceptions when the agreement was moderate, there was substantial to almost perfect agreement between wave quality scores recorded by the two observers with an identical mean value of 4 (i.e. more than one and less than two wavelengths) recorded for both orientations, at all vibration frequencies on both sides of the body. Similarly, and with only one exception when the agreement was poor, there was good to excellent agreement between the measurements of UT stiffness obtained by the two observers for both orientations, at all vibration frequencies on both sides of the body. Thirdly, for both sides of the body, UT stiffness measured when the waves propagated parallel (i.e. VAP) to the principal fibre direction was significantly higher than when the waves propagated orthogonal (i.e. HAP) to the principal fibre direction at all vibration frequencies. In addition, for VAP an expected dispersion effect was observed whereby UT stiffness increased significantly as vibration frequency increased [34]. However, no dispersion effect was observed for HAP. Furthermore, no significant asymmetries in UT stiffness were observed for the left and right sides of the body for either VAP or HAP at any of the vibration frequencies.

In an analysis of trapezius muscle, Ito et al. [5] reported a mean wave quality score of more than 3 for wave images obtained at 50 Hz and 75 Hz, while the mean score obtained at 100 Hz was less than 3. The posterior–lateral positioning of the actuator used by Ito et al was designed to induce wave motion in both supraspinatus and trapezius muscles simultaneously [5], while the VAP and HAP positions were used in the present study to respectively produce wave propagation in two different directions, that were parallel and orthogonal to the principle muscle fibre direction in UT. Secondary waves, that might be generated from the spine of the scapula and which potentially caused wave interference at higher frequencies in the study performed by Ito et al. [5], were not observed in the present study, and this could be the reason for the higher wave quality scores obtained in the present study.

The finding in the present study that the measurement of UT stiffness obtained for waves propagating along the direction of the muscle fibre (i.e. VAP) is higher than when measured for waves propagating orthogonal to the muscle fibre direction (i.e. HAP) is consistent with the results of several previous studies [11, 12, 35, 36]. However, all previous MRE studies of UT used an actuator position which produced the same direction of wave propagation as for VAP [2, 3, 4, 5]. The significant dispersion effect observed in the present study, whereby stiffness values increased as vibration frequency increased for waves propagating parallel to the principle muscle fibre direction (i.e. VAP) is consistent with the results of two previous studies [5, 37]. However, the fact that no significant dispersion effect was obtained for waves propagating orthogonal to the principle muscle fibre direction (i.e. HAP) is a somewhat novel finding in MRE studies. Absence of a dispersion effect for waves propagating in the direction orthogonal to the principle muscle fibre direction was also reported in an ex vivo US‐SWE study of bovine skeletal muscle [38].

With regard to choice of vibration frequency, in general the choice is based on obtaining an optimal balance between adequate shear wave propagation at the chosen depth and good resolving power of the corresponding wavelength [39]. Since UT is a not a deep muscle, relatively high vibration frequencies can be used, such as the value of 150 Hz used Chen et al. [4] to successfully detect taut bands in patients with myofascial pain syndrome. Ito et al. [5]. used a vibration frequency of 75 Hz and reported that the stiffness of UT was 7.26 ± 2.13 kPa for five young male subjects. This is similar to the values of 6.38 ± 1.92 kPa, and 7.19 ± 2.97 kPa, obtained at 80 Hz for left, and right UT, respectively for the nine healthy participants recruited to the present study. However, no evidence was obtained in the present study to suggest that there is any advantage in using a vibration frequency above 60 Hz or wave propagation induced in any direction other than parallel to the principle direction of the muscle fibres. The mean stiffness of 4.25 kPa, with LoA of 1.33 kPa and CV of 22.5% for obtained for left UT, and mean stiffness of 4.22 kPa, with LoA of 1.38 kPa and CV of 33.1%, obtained for right UT at 60 Hz with the actuator in the VAP position (see Table 1) serve as reference values that can be used in future studies.

No significant difference was observed in the stiffness of UT muscle on the left and right sides of the body. Although there are no previous reports of MRE having been used to measure the stiffness of UT on both sides of the body, four studies have been performed using US‐SWE that are relevant. In three studies [40, 41, 42], UT stiffness was reported to be greater on the dominant compared to the non‐dominant side of the body and in one study, similar to the present study, no significant difference was reported between the two sides [43]. In all four SWE studies, measurements were obtained with participants seated on a chair with the shoulder at 0° of abduction and in a neutral position with respect to rotation. This set‐up is very different from the present study where the participants lay in the supine position. Kocur et al. performed SWE of the sternocleidomastoid muscle in the neck in both seated and supine positions [44] and muscle stiffness was found to vary depending upon the position in which participants were measured.

The present study has a number of limitations that should be noted. Firstly, investigations were performed for a relatively small cohort of nine healthy participants, and due to the limited number of participants, separate analysis could not be performed to study the effect of age and sex. In previous studies of cohorts of healthy participants it has been reported that no significant differences in UT stiffness were observed between females and males [40, 45, 46]. Secondly, wave propagation produced by VAP and HAP was considered to be along and orthogonal to the principal muscle fibre directions, respectively. In future studies, waveguide‐constrained MRE utilising diffusion tensor imaging (DTI) [47] could be used to accurately determine the fibre orientation and a new MR inversion algorithm like the heterogeneous model‐based transverse anisotropic implementation of subzone‐based nonlinear inversion (TI‐NLI) [48] could be applied to refine the accuracy of the measurement of UT stiffness in this direction, although longer acquisition times are a potential drawback. Thirdly, UT is a superficial muscle in which vibrations may be readily induced using the soft and flexible tube‐based actuator. However, care has to be taken to ensure that the taping is applied in such a way that the actuator is not pressing too hard against the skin so as to distort the muscle. Fourthly, Leclerc et al. [49, 50] have analysed the mechanical behaviour of the round drum‐type actuator that is provided by Resoundant as part of the clinical product for performing MRE studies of the liver. They demonstrated that between 60 and 100 Hz the membrane of the actuator exhibits one vibration mode and in addition that wave propagation was best at 60 Hz. In the present study potential changes in the mode of vibration were not investigated and could have influenced the stiffness values obtained at the different vibration frequencies. Fifthly, Chen et al. and Ito et al. used different actuators and which were both different to the actuator which has been used in the present study and, including for the reason just discussed, caution should be exercised in comparing the findings of different studies. Sixthly, the viscoelastic properties of UT were not analysed in the present study. In future, MRE data could be acquired at several different vibration frequencies [51] for the purpose of measuring the viscosity of UT muscle using a rheological model [52, 53]. Seventhly, by applying the caliper technique MRE studies of muscle can be performed taking into account the inherent anisotropic properties of muscle. However knowing that they would contain biases, in the present study an analysis was not performed for the elastograms that had been produced by the MMDI inversion algorithm. In future work it will be interesting if algorithms can be developed so that elastograms and associated confidence maps could be produced based on the so‐called phase gradient technique [15] and which would allow the direction of the muscle fibres to be taken into account in performing detailed mapping of muscle stiffness. Lastly, a 2D MRE pulse sequence was used in the present study. 2D MRE may be considered an ideal approach when it is known that wave propagation is oriented along the fibre direction of the muscle and when this direction lies within a 2D plane. The latter may be a more reasonable assumption for studying fusiform muscles such as rectus femoris in the thigh than for UT. In future, 3D MRE should be used for measuring the stiffness of UT muscle.

The motivation for performing the present study was to develop a muscle MRE protocol that can be used to provide an objective outcome measure of muscle stiffness in a randomized control trial to compare the efficacy of Thai traditional massage with more focussed physical therapy for treating office syndrome. In particular, physical therapy treatments require the use of specialised ultrasound equipment for disrupting myofascial trigger points in UT, complemented by use of passive stretching and heat therapy to release taut bands, whereas Thai traditional massage is a practical, convenient, widely available and popular choice for self‐referral to treat office syndrome in Thailand, the benefit of which is well accepted but still to be proven scientifically. The caliper technique offers a simple and convenient approach for these investigations with UT stiffness obtained by analysing wave images produced by 2D GRE MRE. Furthermore, the new soft and flexible tube‐based actuator is comfortable and produced very good wave propagation in UT and is recommended to be used with a vibration frequency of 60 Hz, which is the standard value used in clinical MRE studies of liver, and oriented so that wave propagation is induced in the direction parallel to the principle direction of the muscle fibres.

## Data Availability

The data that support the findings of this study are available from the corresponding author upon reasonable request.
